# Potential‐Dependent Reversible Restructuring of Pt–Au Alloy Electrocatalysts

**DOI:** 10.1002/smll.74916

**Published:** 2026-07-28

**Authors:** Jan Sebastian Dominic Rodriguez, Hassan Javed, Kees E. Kolmeijer, Xianxian Xie, Athira Lekshmi Mohandas Sandhya, Marijn A. van Huis, Ivan Khalakhan, Cheng‐Hao Chuang, Rik V. Mom

**Affiliations:** ^1^ Leiden Institute of Chemistry Leiden University Leiden The Netherlands; ^2^ Department of Physics Tamkang University New Taipei City Taiwan; ^3^ Department of Surface and Plasma Science Faculty of Mathematics and Physics Charles University Prague Czech Republic; ^4^ Debye Institute for Nanomaterials Science Utrecht University Utrecht The Netherlands

**Keywords:** alloy, electrocatalyst, electrochemistry, materials science, nanoparticle, x‐ray photoelectron spectroscopy

## Abstract

The distribution of metals in alloy nanoparticles is a key parameter in their electrochemical performance. Here, we show that for Pt–Au alloys this distribution dynamically responds to the electrochemical environment. Using electrochemical X‐ray photoelectron spectroscopy, we find that the surface composition of the alloy can range from stoichiometric to Pt‐enriched, depending on the applied potential. The driving factors for this restructuring are the differences in electronegativity and mobility between Pt and Au. At potentials below 1.5 V_RHE_, the slightly more reactive (less electronegative) Pt shows surface enrichment, which slowly increases as the potential is raised and increasingly oxidizing adsorbates are formed. Above 1.5 V_RHE_, oxidation of the more mobile Au atoms becomes possible, which is kinetically favored over Pt oxidation, thereby driving Au toward the surface. All surface dynamics are found to be highly reversible, even at room temperature. This facile rearrangement suggests that dynamic restructuring of alloy nanoparticles occurs in a wide range of conditions, and should therefore be taken into account in alloy electrocatalyst design.

## Graphical abstract

Using electrochemical X‐ray photoelectron spectroscopy (XPS), we show that Pt–Au alloys adjust their surface elemental composition to the applied electrochemical conditions. The surface dynamics are found to be highly reversible, even at room temperature, suggesting that dynamic restructuring of alloy nanoparticles occurs in a wide range of conditions, and should therefore be taken into account in alloy electrocatalyst design.

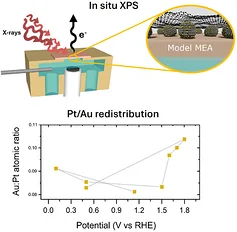

## 1 Introduction

Platinum is a cornerstone metal in electrocatalysis, showing high performance in a wide range of electrochemical reactions, including the hydrogen evolution reaction (HER), oxygen reduction reaction (ORR), and methanol oxidation reaction. To reach optimal performance in these different reactions, platinum is often alloyed with metals such as Co [1, 2], Ni [3], Au [4], and Ru [5]. These alloying metals either directly participate in the catalysis by providing alternative active sites for bottleneck reaction steps, or they indirectly influence the catalysis by modulating the electronic structure of the active Pt atoms via ligand and strain effects.

In both the direct and indirect mechanisms, the position of the alloying metal in the catalyst particles plays a crucial role in the performance enhancement. For example, surface Ni atoms are essential for promoting water dissociation in Pt‐Ni catalysts for the alkaline HER [3, 6]. Similarly, for the alloy core/Pt shell catalysts used in fuel cells, the thickness of the Pt shell has a marked effect on the ORR activity. For thinner Pt shells, the Ni or Co atoms in the core of the particle exert stronger ligand and strain effects on the catalytic Pt surface atoms, which (favorably) weakens the binding strength of the oxygen intermediates [7].

Given that the configuration of alloys thus plays an important role in the activity of the electrocatalyst, a key question that arises is whether the catalyst structure changes under electrochemical conditions. For several alloys, such changes have indeed been identified. For example, acidic electrolytes induce leaching of Ni and Co in Pt‐Ni and Pt‐Co alloys [8, 9], forming a (rough) pure Pt shell at the surface while the alloy remains in the bulk [10]. While this can lead to higher surface area, the lower amount of Ni/Co atoms at the surface and subsurface results in a decreased degree of strain and ligand effect, thus resulting in decreased ORR activity [10]. For more noble alloying metals such as Ag and Cu, leaching also occurs, but only under (oxidizing) applied potential [11, 12]. Aside from leaching, other in situ induced restructuring phenomena such as bulk phase transformations [13, 14] and Ostwald ripening can further move the alloy's structure from the as‐prepared state, with significant impact on their performance [8, 9, 15, 16, 17, 18.

Recently, we found that on top of this irreversible alloy restructuring, alloys can also restructure reversibly upon changes in applied potential [19]. Specifically, we showed for a Pt_3_Ni core/Pt shell alloy that Ni moves reversibly between the core and the near‐surface of the alloy. We identified charge transfer from the (non‐noble) Ni atoms to the adsorbates on the surface as the driving force for this restructuring: higher potentials lead to more oxidized, electron‐withdrawing adsorbates, which triggers this charge transfer and draws Ni closer to the surface.

Based on this identified mechanism, reversible alloy restructuring should be a phenomenon that primarily occurs in alloys between metals with large differences in electronegativity, such as the combination of a noble and a non‐noble metal, where charge transfer from the non‐noble metal to the adsorbates on the catalyst surface modulates their position in the catalyst. To test whether this is indeed the case, or whether other phenomena also play a role in reversible alloy restructuring, we here study a Pt–Au alloy, i.e., a material where the alloying element is slightly more noble than the Pt host. Using electrochemical X‐ray photoelectron spectroscopy (XPS), we tracked the (near‐)surface composition of the alloy as a function of potential. We show that over a wide potential range, the alloy indeed shows only limited restructuring, as expected from the small difference in electronegativity. However, above the Au oxidation potential, we observe a rapid increase of Au on the surface. We identify the higher mobility of Au compared to Pt as the likely source of this effect.

## 2 Methods

### 2.1 Electrochemical XPS

The electrochemical XPS measurements were performed in our laboratory‐based near‐ambient‐pressure XPS (NAP‐XPS) setup, which is equipped with a custom‐designed spectro‐electrochemical flow cell that can accommodate a membrane electrode assembly (MEA) (Figure 1). The details of the setup and cell construction were discussed in our previous work [20]. In short, the MEA consists of a ∼5 nm Pt_9_Au_1_ nanoparticle catalyst layer sandwiched between a Nafion membrane and a bilayer graphene (sample preparation details in the Supporting Information). This composition mimics a fuel cell catalyst layer: the graphene layer models the carbon support, the alloy particles the catalyst, and the Nafion membrane the ionomer. However, in our spectroelectrochemical cell, these components have additional functions. The Nafion membrane allows permeation of the electrolyte in order to wet the catalyst. Meanwhile, the bilayer graphene serves as a window that minimizes evaporation of electrolyte into the chamber. In previous work, we have shown that the graphene barrier is sufficient to maintain a liquid film around the catalyst [21]. Meanwhile, the transparency of the graphene window allows X‐rays to come in and photoelectrons to escape, while graphene's conductivity ensures electrical contact to the catalyst.

**FIGURE 1:**
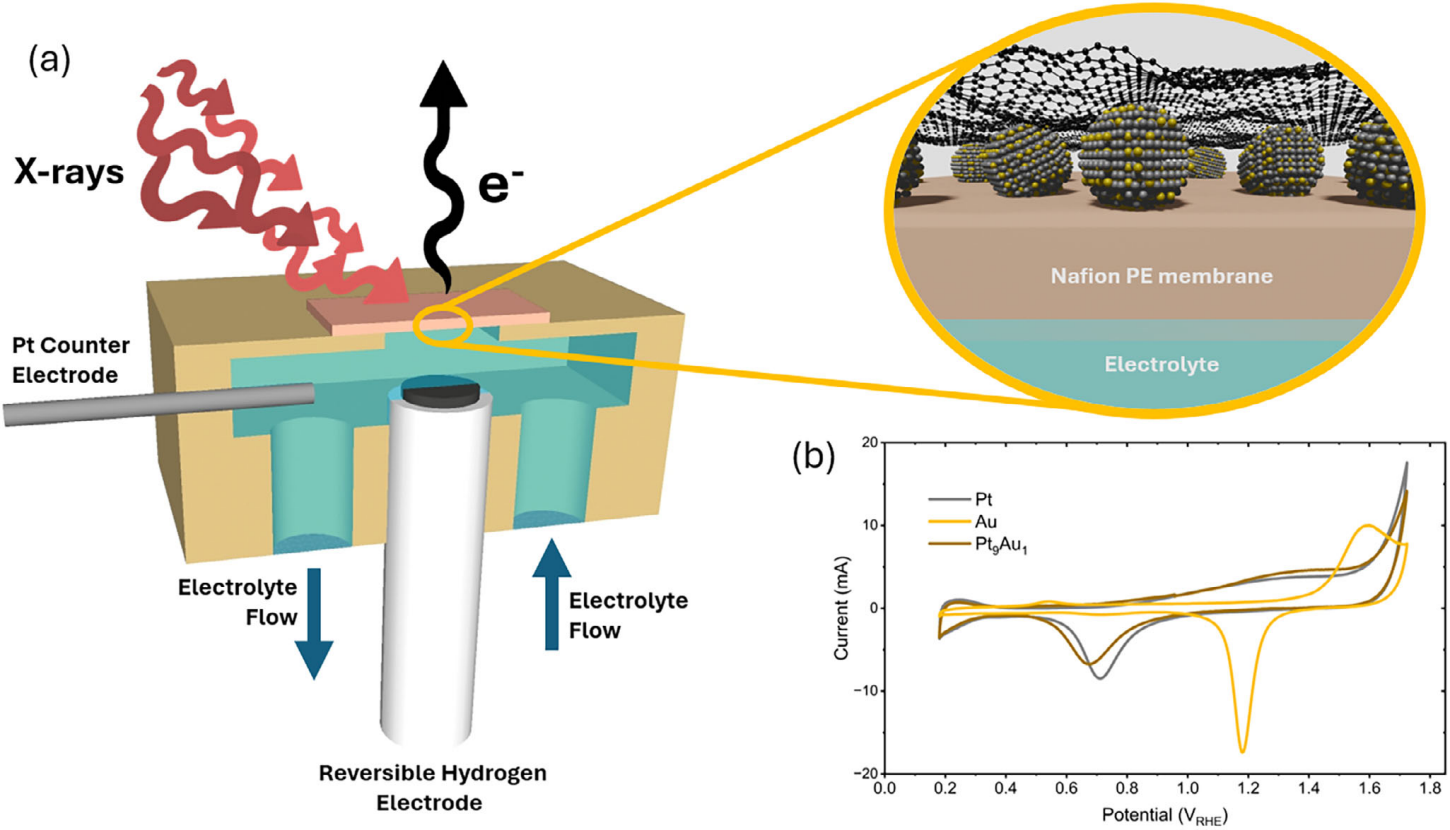
(a) Electrochemical XPS cell fitted with an MEA supporting the Pt–Au alloy catalyst. (b) Cyclic voltammograms (CVs) of pure Pt, pure Au, and Pt_9_Au_1_ MEA electrocatalysts recorded in the electrochemical XPS cell in 0.1 M HClO_4_ at 20 mV/s. The current of each CV is normalized to its corresponding double‐layer current value to facilitate comparison.

A SPECS µFOCUS 600 Monochromated Al Kα X‐ray Source is used in the NAP‐XPS setup, which has an X‐ray energy of 1486.7 eV. This allowed us to study both the Pt and the Au atoms with a photoelectron attenuation length of ∼1.3 nm [22]. The XPS signal was collected by a SPECS PHOIBOS 150 analyzer. Due to the risk of beam damage to the Nafion membranes after an extended period of exposure, each spectrum was recorded at a new spot on the surface, exploiting the inherent homogeneity of sputter‐coated metal films on flat substrates. This homogeneity is verified based on the noise and reproducibility of the trends observed in the analyzed data. The XPS data were processed using the CASA XPS software. Details of the XPS fitting and data analysis are available in the Supporting Information.

During the electrochemical XPS measurements, the working electrode potential was controlled with a Biologic SP‐200 potentiostat, connected to the MEA, a Pt counter electrode, and a reversible hydrogen reference electrode (Gaskatel Hydroflex) in the cell. Prior to the spectroscopy measurements, the potential was cycled between 0.05 and 1.2 V_RHE_ at 50 mVs^−1^ to condition the sample and check its proper electrochemical behavior. During the spectroscopy measurements, the following potential sequence was used: 1.14, 0.1, 0.5, 1.5, 1.6, 1.7, and 1.8 V_RHE_. With this sequence, the potential is moved up and down repeatedly in order to verify reversibility and reproducibility of data.

### 2.2 Transmission Electron Microscopy

For the transmission electron microscopy (TEM) measurements, PELCO Au TEM grids with support films of Formvar/Carbon were dip‐coated with a thin Nafion film in a resin solution (5 wt.% Nafion in lower alcohols, Sigma–Aldrich) which was diluted 10× in ethanol. The Nafion coating was applied so that an equivalent nanoparticle structure would be formed during sputter coating as for the electrochemical XPS samples. The nanoparticle/Nafion‐coated TEM grids were analyzed using electron microscopy, using a TFS Spectra300 TEM, equipped with a monochromator and double aberration correction, and operated at 300 kV in STEM (scanning transmission electron microscopy) imaging mode with a semi‐convergence angle of 20.2°. For STEM‐HAADF (high‐angle annular dark field) imaging, a probe current of 0.2 nA and a dwell time of 10 µs were used. Chemical mapping was performed using STEM‐EDS (energy dispersive X‐ray spectroscopy) measurements, where a probe current of 1.6 nA and a dwell time of 2.5 µs were used while the region of interest was scanned continuously for 6–8 min to acquire the Pt and Au compositional maps.

## 3 Results

To create a well‐defined Pt–Au nanoparticle alloy sample with a uniform response to the electrochemical environment, we prepared a model MEA consisting of a monolayer of sputter‐deposited Pt_9_Au_1_ nanoparticles on a Nafion substrate (see Section 2). Figure 2 shows the resulting nanoparticle film structure, with an average grain size of 5.2 ± 2.1 nm (size distribution analysis in Figure S1). The as‐prepared Pt/Au distribution in the particles is not fully homogeneous, showing some Au surface enrichment, particularly at particle edges and grain boundaries. Enrichment at these undercoordinated sites may be related to the lower surface energy of Au in vacuum (i.e., in the absence of adsorbates) compared to that of Pt [23, 24].

**FIGURE 2:**
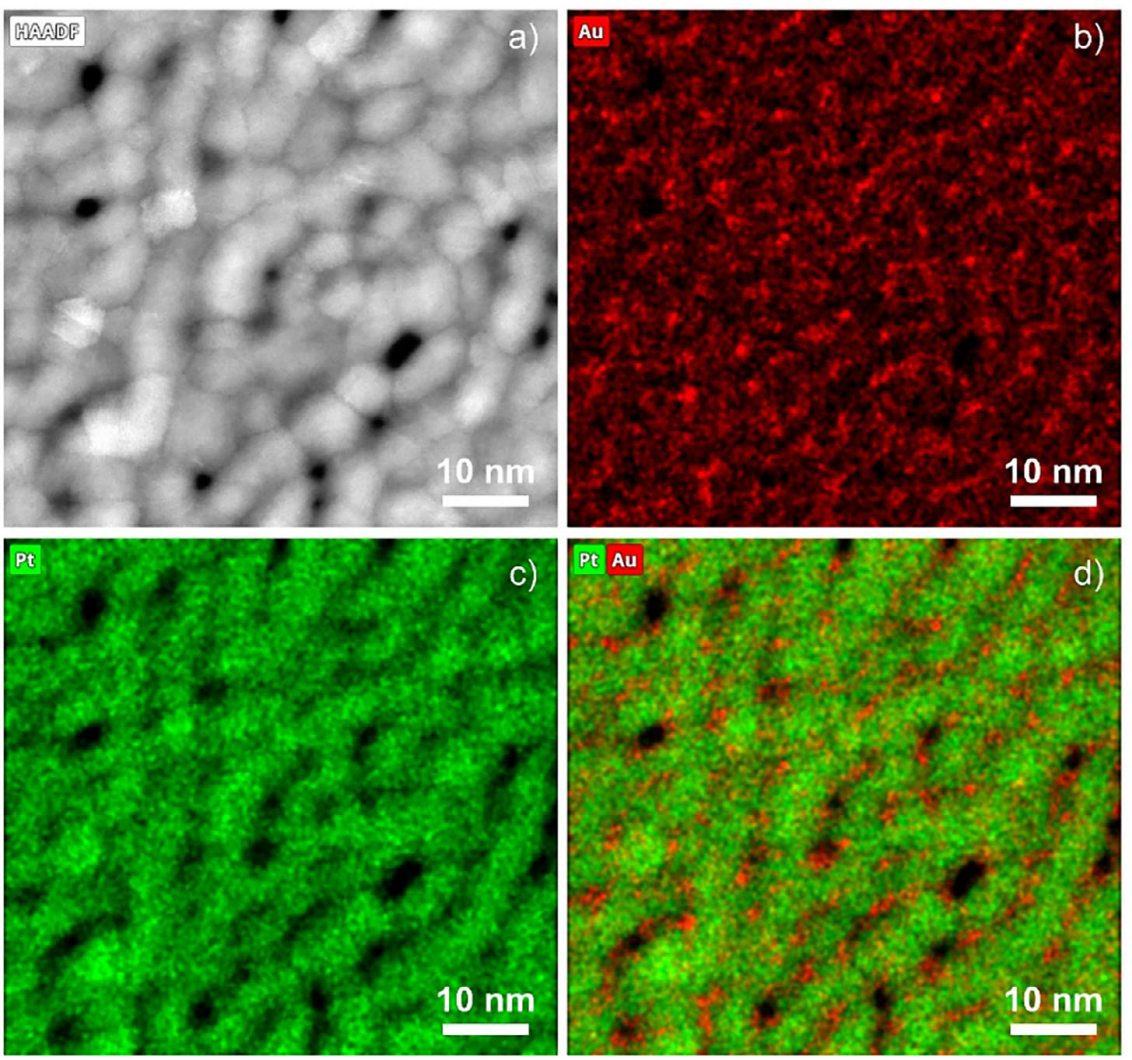
(a) High‐resolution STEM‐HAADF image of Pt_9_Au_1_ nanoparticle film supported on Nafion‐coated TEM grid. (b–d) STEM‐EDS images showing elemental maps of Au, Pt, and Au+Pt overlay, respectively.

Following nanoparticle film preparation, the Nafion‐supported particles were covered with a graphene window for the spectro‐electrochemical studies (Figure 1a). An initial assessment of the surface chemistry of this Nafion‐Pt_9_Au_1_‐graphene assembly was made using cyclic voltammetry (CV) (Figure 1b). When comparing the voltammogram of Pt_9_Au_1_ to that of Pt and Au samples prepared in the same manner, it is clear that the alloy's electrochemical signature predominantly resembles that of Pt. This provides a first indication that the Au enrichment observed for the as‐prepared particles is not sustained under electrochemical conditions.

To further track the chemistry of the Pt_9_Au_1_ alloy, electrochemical XPS measurements were conducted at a series of applied potentials. Au 4f and Pt 4f spectra (Figure 3a,b) were recorded over a potential range in which the surface is expected to transition from an H‐covered metallic state all the way to a surface oxide. The Au 4f spectra were fitted using doublets for Au^0^, Au^int.^, and Au^3+^, with binding energy ranges determined using the literature [25, 26, 27] and reference data for the electrochemical oxidation of pure Au (see Section S2). Note that Au^int.^ refers to Au atoms at the interface between (surface) oxide and metal. Its surprisingly low binding energy was previously observed for pure Au oxidized in ozone [26], and is also found in our reference data for pure Au. Pt 4f spectra were fitted using Pt^0^, Pt^δ+^, Pt^2+^ and Pt^4+^ doublets, adopting a fitting model established for pure Pt in previous work [20, 21]. Additional details of the fitting parameters are available in the Supporting Information.

**FIGURE 3:**
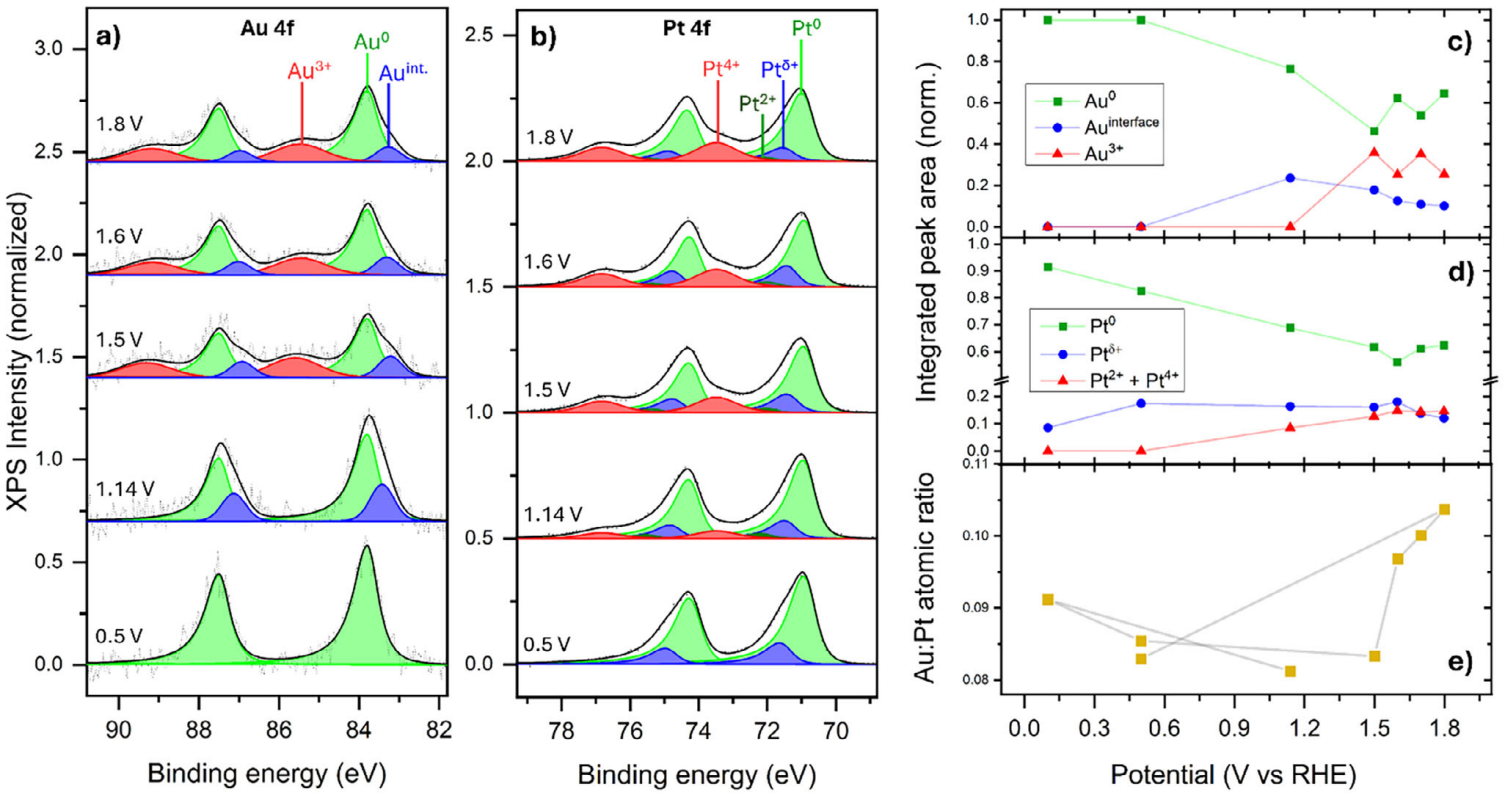
XPS spectra of (a) the Au 4f and (b) the Pt 4f core levels, acquired from the PtAu alloy MEA; (c) peak areas of Au^0^, Au^interface^, and Au^3+^, and (d) Pt^0^, Pt^δ+^, and Pt oxides, as functions of potentials; (e) atomic ratio trend of Au and Pt. The parameters used for the spectral fitting of the Au 4f and Pt 4f peaks are provided in Section S2.

From the fitting of the data, we extracted the evolution of the oxidation states of the Pt and Au atoms as a function of potential (Figure 3c,d). The contributions of the oxidized states of Pt, namely Pt^2+^ and Pt^4+^, were summed together for clarity of presentation. At potentials where neither Au nor Pt are expected to be oxidized (0.1 and 0.5 V_RHE_), Pt^0^ is dominant in the Pt 4f spectra. A Pt^δ+^ contribution is observed, however, which we attribute to the presence of an adsorbate‐induced dipole, similar to previous studies on pure Pt [21]. As expected from the literature (e.g., [28]), Pt is oxidized at potentials above 1.0 V_RHE_, showing an increase in both Pt^2+^ and Pt^4+^ contributions. This mixed oxide formation immediately at the onset of Pt oxidation is in line with previous results on pure Pt [21]. Interestingly, however, no significant increase in the Pt oxides is observed at potentials higher than 1.6 V_RHE_, contrary to what has been found in pure Pt, where the thickness of the PtO_2_ shell steadily increased with increased applied potential up to 1.85 V_RHE_ [21].

In the Au 4f spectra, only Au^0^ is observed at low potential (0.1 and 0.5 V_RHE_). The lack of an adsorbate‐induced dipole peak like the Pt^δ+^ component seen for Pt is in agreement with the more noble, weak‐binding character of Au compared to Pt. Indeed, our reference spectra for pure Au also showed only Au^0^ at low potential (see Section S2). At 1.14 V_RHE_, where the Pt surface atoms oxidize, an Au^interface^ contribution can be identified. This hints at Pt oxides in contact with Au atoms in the PtAu alloy, verifying the presence of Au at the (near‐)surface. At higher potentials (1.5 V_RHE_ and above), an Au^3+^ peak is observed arising from the oxidation of Au [26, 29, 30]. The onset potential for Au oxidation is in line with the of Au (Figure 1), where a sharp Au oxidation onset at 1.5 V_RHE_ is observed.

As indicated previously, the Au oxidation/reduction features are not clearly observed in the CV of the Pt_9_Au_1_ particles, which may be interpreted as an indication that the particle surface is dominated by Pt. To further investigate the surface elemental composition of the particles and its response to the chemical changes of the surface discussed above, we tracked the Au 4f/Pt 4f intensity ratio as a function of potential. After correcting for the X‐ray photoionization cross‐sections of the Au 4f and Pt 4f core levels and considering that the probing depths for these core levels are the same [31], this ratio provides a direct measure of the atomic ratio within the probing depth of the XPS measurement. Using Shard's model [32], we can estimate this probing depth given the particle size observed in STEM‐HAADF: ∼80% of the signal originates from the top 1 nm. At 0.1 V_RHE_, it can be observed that the surface is slightly enriched with Pt, marginally exceeding the Pt_9_Au_1_ bulk composition. This indicates that the adsorbed H_ads_ present at this potential does not strongly favor either Au or Pt. When increasing the potential, a gradual small decrease in the Au:Pt ratio is observed in the potential range up to 1.13 V_RHE_, indicating a slight increase in the Pt surface enrichment. This is in line with the model we recently derived for a PtNi alloy [19]: when the potential is increased, the adsorbate structure on the surface changes (e.g., H_ads_ to H_2_O_ads_ and OH_ads_), becoming increasingly electron‐withdrawing. The less noble metal in the alloy, in this case Pt, provides this electron density, creating an adsorbate‐Pt dipole that draws the Pt to the surface in analogy to the Cabrera‐Mott model [33]. Indeed, Figure 3d shows a gradual decrease of Pt metallicity over this potential range, supporting the idea of electron transfer to adsorbates. The observation of a (slightly) Pt‐enriched surface is also well in line with dominant Pt features in the CV in Figure 1b.

Above 1.5 V_RHE_, the trend of increasing Pt enrichment with increasing potential is broken. The amount of Au atoms in the (near‐) surface drastically increases, with a continuing upward trend to 1.8 V_RHE_. This means that not only the less noble Pt atoms are drawn to the surface, but the more noble Au. This transition from Pt migration to Au migration coincides with the Au oxidation potential. As can be seen from Figure 3c,d, once Au oxidation is possible, it is preferred over Pt oxidation, which stagnates at 1.5 V_RHE_ and above. This preference can be attributed to the higher mobility of Au compared to Pt atoms in a PtAu alloy system [34]. At 1.5 V_RHE_, the top monolayer of the surface is oxidized [35]. Continued oxidation deeper into the material faces a significant kinetic barrier for Pt due to low Pt atom mobility. The higher mobility of Au therefore makes it kinetically favored. Thus, we identify mobility as a second key factor in alloy restructuring, in addition to the electronegativity of the alloy components.

Note that all restructuring discussed above proved reversible, as the Au:Pt atomic ratio returned to its previous value when the applied potential was moved up and down (see sequence from Figure 3e). By comparing the CV response to the XPS response, we can get a sense of the time scale of the Pt/Au movement within the particles. For the XPS measurements, which occur on the time scale of ∼20 min per potential, we are able to observe clear reversible restructuring. In contrast, Au is not sufficiently drawn to the surface by high potential (> 1.5 V_RHE_) to yield an appreciable Au oxidation/reduction signal in the Pt_9_Au_1_ CV, which is measured on the time scale of seconds. Overall, these observations show that there are mild kinetic restrictions to the alloy restructuring, but that it is sufficiently facile to reach a (quasi‐)equilibrium state on the minute time scale, even at room temperature. Since we observed this for an alloy with a low driving force for restructuring (small electronegativity difference) and under mild conditions, one may speculate that dynamic alloy restructuring is a widespread phenomenon in alloy‐based electrocatalysis.

## 4 Conclusion

Using electrochemical XPS, we have tracked the surface composition and oxidation state of a Pt_9_Au_1_ nanoparticle alloy. We find that the surface Au:Pt ratio dynamically responds to the applied potential, ranging from a Pt‐enriched to a stoichiometric state. The restructuring is strongly correlated to the surface chemistry of the alloy. When the surface is metallic, it is slightly enriched with Pt, which is more reactive than Au and therefore has stronger interactions with adsorbates. For more oxidizing adsorbates and Pt surface oxide, this effect is stronger than for the adsorbed hydrogen present at low potentials. Above 1.5 V_RHE_, when bulk oxidation of both Pt and Au becomes possible, metal mobility becomes the key factor. The higher mobility of Au promotes its oxidation kinetics, thereby driving Au toward the surface. Importantly, we find that all alloy restructuring is highly reversible, implying that the alloy is able to reach a (quasi‐)equilibrium state at every potential, even at room temperature. As Pt–Au alloys are intermediate‐melting‐point alloys with a comparatively low driving force for restructuring (small difference in electronegativity between Pt and Au), the facile restructuring under mild conditions observed here suggests that potentiodynamic alloy restructuring is likely a widespread phenomenon in alloy electrocatalysis. We therefore expect that taking potentiodynamic restructuring into account will be important for accurate prediction of alloy electrocatalyst performance.

## Author Contributions

**Ivan Khalakhan**: resources, supervision. **Athira Lekshmi Mohandas Sandhya**: investigation. **Xianxian Xie**: investigation. **Jan Sebastian Dominic Rodriguez**: investigation, visualization, writing – original draft, formal analysis. **Hassan Javed**: investigation, visualization, formal analysis. **Marijn A. van Huis**: investigation, resources. **Kees E. Kolmeijer**: investigation, methodology. **Cheng‐Hao Chuang**: project administration, resources, funding acquisition, supervision. **Rik V. Mom**: resources, supervision, writing – review and editing, validation, funding acquisition, conceptualization.

## Conflicts of Interest

The authors declare no conflicts of interest.

## Supporting information




**Supporting File**: smll74916‐sup‐0001‐SuppMat.docx.

## Data Availability

The data that support the findings of this study are available from the corresponding author upon reasonable request.
