# Supplementary material for: Potential‐Dependent Reversible Restructuring of Pt–Au Alloy Electrocatalysts
Source: Small. 2026 Jul 28;22(55):e74916. doi: 10.1002/smll.74916 (PMC13630412; doi:10.1002/smll.74916)
Supplement: Supplementary file 1 [file SMLL-22-e74916-s001.docx]

**Supporting information for**

**Potential-dependent reversible restructuring of Pt-Au alloy electrocatalysts**

Jan Sebastian Dominic Rodriguez^1,2†^, Hassan Javed^1†^, Kees E. Kolmeijer^1^, Xianxian Xie^3^, Athira Lekshmi Mohandas Sandhya^3^, Marijn A. van Huis^4^, Ivan Khalakhan^3^_,_ Cheng-Hao Chuang^2^, Rik V. Mom^1*^

^1^Leiden Institute of Chemistry, Leiden University, Leiden 2300 RA, The Netherlands

^2^Department of Physics, Tamkang University, New Taipei City, Taiwan

^3^Department of Surface and Plasma Science, Faculty of Mathematics and Physics, Charles University, 1800 Prague 8, Czech Republic

^4^Debye Institute for Nanomaterials Science, Utrecht University, Princetonplein 5, 3584 CC Utrecht, The Netherlands

[*r.v.mom@lic.leidenuniv.nl](mailto:*r.v.mom@lic.leidenuniv.nl)

† Authors contributed equally

**S1	Sample preparation for electrochemical XPS**

Nafion 117 membrane was cleaned in a 3% H_2_O_2_ solution at 80^o^C for 2 hr and then activated in 0.5 M H_2_SO_4_ solution, again at 80^o^C for 2 hr. Following the cleaning procedure, the membranes were rinsed with deionized water and dried before sputter coating with electrocatalyst nanoparticles. In the sputter coating process, ~5 nm films of Pt, Au and Pt_9_Au_1_ particles were deposited onto the Nafion membranes using two magnetrons (Kurt J. Lesker) able to operate simultaneously. To this end, 2" Pt (99.99% Safina) and 2" Au (99.99% Kurt J. Lesker) targets were employed. The Pt:Au ratio was calibrated using an EDX calibration series (including thicker films) and benchmarked with other characterization methods.^1^ The error margin is estimated to be no more than ±10% (relative).

Following the preparation of the membrane-electrode assembly (MEA), a graphene bi-layer window was loaded on top of the Pt/Au/Pt-Au nanoparticles to impede electrolyte evaporation into the XPS vacuum chamber during the spectroscopic measurements. The graphene window has a high transparency towards both X-rays and photoelectrons. In addition, it provides the electrical contact to the electrocatalyst nanoparticles. To load the graphene onto the MEA, graphene supported on copper (General Graphene) was floated on a 40 g/L ammonium persulfate etching solution overnight, dissolving copper and leaving the graphene layer floating on the liquid surface. The solution was then exchanged with pure water and the floating graphene was scooped off the surface of the water with the MEA. The prepared sample with graphene was dried at room temperature to facilitate proper graphene adhesion and stored until use in the electrochemical XPS experiments.

The grain size distribution was estimated from HAADF-STEM images. A histogram of the size distribution is provided in Figure S1, yielding an average grain size of 5.2 ± 2.1 nm.

**Figure S1.** Grain size distribution of a collection of 163 grains. The grain sizes were measured manually in HAADF-STEM images using ImageJ software. The average size and imprecision interval of 5.2 ± 2.1 nm are derived from the statistical distribution. The actual uncertainty is larger because of the irregular shapes of the grains (see Fig. 2a in the main text).

**S2	XPS Data fitting**

The XPS data was analyzed using CasaXPS Version 2.3.23. A Tougaard background subtraction was used for the Au 4f region, while a Shirley background subtraction was used for the Pt 4f region. To fit the Pt 4f region, we employed a fitting model established in previous work^2,3^Click or tap here to enter text.. The model uses an asymmetric Gaussian-Lorentzian LF line shape that is characterized by four parameters: α, β, w and m. Here, α and β characterize the Lorentzian tail spreading of the low and high binding energy half of the peak, respectively, allowing tuning of the asymmetry level. The parameter m sets the level of Gaussian convolution applied to the Lorentzian function. Finally, w is the dampening factor applied to the Lorentzian tail far from the peak center. Doublets for Pt^0^ Pt^δ+^, Pt^2+^ and Pt^4+^ with a constant spin-orbit splitting of 3.34 eV and a peak area ratio of 3:4 for the 4f_5/2_ and 4f_7/2_ peaks were used. The binding energies of the components were taken from previous work^2,3^ ^1,2^, and are in good agreement with the literature.^43^ The details of the fitting parameters are shown in Table S1.

|  | **Pt^0^** | **Pt^δ+^** | **Pt^2+^** | **Pt^4+^** |
| --- | --- | --- | --- | --- |
| **Line shape** | LF(0.9,2,60,50) | LF(0.9,2,60,50) | LF(0.9,2,60,50) | LF(3,3,30,10) |
| 4f_7/2_ Peak Position | 71.2 - 70.9 eV | 72 - 71.2 eV | 72.7 – 72 eV | 73.7 eV |
| FWHM | Free | Same as Pt^0^ | Same as Pt^0^ | 1.44 eV |

**Table S1**: Fitting parameters for the Pt 4f spectral region

To fit the Au 4f data, we first established a basic fitting model for pure Au nanoparticles prepared in the same type of Nafion-nanoparticle-graphene MEA as the alloy. The data were recorded at the ISISS beamline at BESSY II, Berlin, Germany with an X-ray energy of 600 eV. The fitted data is displayed below in Figure S2. Already by eye, it is clear that the data can be fitted using three Gaussian-Lorentzian doublets. The green component is assigned to Au^0^, while the red component is assigned to Au^3+^, consistent with the literature^5–7^Click or tap here to enter text.. A remarkable low-binding energy component is also clearly visible in the data. This component was also observed during the oxidation of Au using ozone^5^Click or tap here to enter text.. We attribute this component to metallic Au atoms that are in contact with the Au oxide layer.

Au^0^

Au^int^

Au^3+^

**Figure S2:** Electrochemical XPS study of graphene-covered pure Au nanoparticles on Nafion. Indicated potentials are on the RHE scale. OCP refers to open circuit potential.

With the basic fitting components established, we adapted the fitting model to our laboratory XPS instrument, which has a lower spectral resolution and therefore yields somewhat more Gaussian peaks. A LA lineshape was used with a spin-orbit splitting of 3.704 eV and a 4:3 ratio for the Au 4f_7/2_ and 4f_5/2_ peaks. The details of the fitting parameters are shown in Table S2.

|  | **Au^0^** | **Au^int^** | **Au^3+^** |
| --- | --- | --- | --- |
| **Line shape** | LA(1,1.6,0) | LA(1,643) | LA(1,643) |
| 4f_7/2_ Peak Position | 84.0 eV | Free  (83.2-83.3 eV) | Free  (85.5-85.6 eV) |
| FWHM | Determined iteratively to be 0.649 | Determined iteratively to be 0.707 | Determined iteratively to be 1.5 |

**Table S2**: Fitting parameters for the Au 4f spectral region

**References**

(1)	Xie, X.; Briega-Martos, V.; Farris, R.; Dopita, M.; Vorokhta, M.; Skála, T.; Matolínová, I.; Neyman, K. M.; Cherevko, S.; Khalakhan, I. Optimal Pt–Au Alloying for Efficient and Stable Oxygen Reduction Reaction Catalysts. *ACS Appl. Mater. Interfaces* **2023**, *15* (1), 1192–1200. https://doi.org/10.1021/acsami.2c18655.

(2)	Javed, H.; Knop-Gericke, A.; Mom, R. V. Structural Model for Transient Pt Oxidation during Fuel Cell Start-up Using Electrochemical X-Ray Photoelectron Spectroscopy. *ACS Appl. Mater. Interfaces* **2022**, *14* (31), 36238–36245. https://doi.org/10.1021/acsami.2c09249.

(3)	Mom, R.; Frevel, L.; Velasco-Vélez, J.-J.; Plodinec, M.; Knop-Gericke, A.; Schlögl, R. The Oxidation of Platinum under Wet Conditions Observed by Electrochemical X-Ray Photoelectron Spectroscopy. *J. Am. Chem. Soc.* **2019**, *141* (16), 6537–6544. https://doi.org/10.1021/jacs.8b12284.

(4)	Saveleva, V. A.; Papaefthimiou, V.; Daletou, M. K.; Doh, W. H.; Ulhaq-Bouillet, C.; Diebold, M.; Zafeiratos, S.; Savinova, E. R. Operando Near Ambient Pressure XPS (NAP-XPS) Study of the Pt Electrochemical Oxidation in H2O and H2O/O2 Ambients. *J. Phys. Chem. C* **2016**, *120* (29), 15930–15940. https://doi.org/10.1021/acs.jpcc.5b12410.

(5)	Klyushin, A. Y.; Rocha, T. C. R.; Hävecker, M.; Knop-Gericke, A.; Schlögl, R. A near Ambient Pressure XPS Study of Au Oxidation. *Phys. Chem. Chem. Phys.* **2014**, *16* (17), 7881–7886. https://doi.org/10.1039/C4CP00308J.

(6)	Peuckert, M.; Coenen, F. P.; Bonzel, H. P. On the Surface Oxidation of a Gold Electrode in 1N H2S04 Electrolyte. *Surface Science* **1984**, *141* (2), 515–532. https://doi.org/10.1016/0039-6028(84)90146-8.

(7)	Krozer, A.; Rodahl, M. X-Ray Photoemission Spectroscopy Study of UV/Ozone Oxidation of Au under Ultrahigh Vacuum Conditions. *J. Vac. Sci. Technol. A* **1997**, *15* (3), 1704–1709. https://doi.org/10.1116/1.580924.
